# Intelligence overload: How AI is rewiring our approach to neurodegenerative diseases

**DOI:** 10.1016/j.bas.2024.102868

**Published:** 2024-07-11

**Authors:** Gokul Sudhakaran

**Affiliations:** Center for Global Health Research, Saveetha Medical College and Hospital, Saveetha Institute of Medical and Technical Sciences (SIMATS), Kancheepuram district, Thandalam, Tamil Nadu, India

Neurodegenerative diseases, such as Alzheimer's and Parkinson's, have long been enigmas, challenging the brightest minds in medicine. However, a new era of research has dawned, thanks to the integration of artificial intelligence (AI) in our investigative arsenals. This letter seeks to highlight the transformative potential of AI in diagnosing, understanding, and treating neurodegenerative diseases.

The cornerstone of effective neurodegenerative disease management is early diagnosis, a task at which AI excels due to its ability to sift through vast datasets faster and precision more precisely than humanly possible. Tools like deep learning algorithms have demonstrated remarkable accuracy in detecting subtle patterns in brain scans that precede symptomatic onset of diseases such as Alzheimer's. Studies have shown that AI can identify biomarkers from neuroimaging data with a precision that outstrips current clinical methods, potentially leading to earlier interventions that could slow disease progression or improve patient outcomes.

Furthermore, AI's capability extends beyond diagnosis into treatment and management. Machine learning models are trained to predict disease progression based on initial symptoms and ongoing patient data. This can aid clinicians in crafting personalized treatment plans that are more likely effective for individual patients, potentially extending quality of life.

On the research front, AI is making strides in elucidating the pathophysiology of neurodegenerative conditions. By analyzing complex biochemical and genetic information, AI systems can identify novel therapeutic targets that would be too subtle or complex for traditional research methods. For instance, AI has been used to model the interaction between genetic factors and environmental influences in Parkinson's disease, offering new insights into causal mechanisms and potential intervention points.

Despite these advancements, the deployment of AI in clinical settings faces significant challenges, including ethical concerns regarding data privacy, the need for extensive validation of AI tools in clinical trials, and the potential for AI to perpetuate existing biases if not carefully managed. Therefore, while AI presents a promising tool in our fight against neurodegenerative diseases, it must be implemented thoughtfully and in concert with traditional medical expertise.

In conclusion, as we stand on the brink of what could be a revolution in neurodegenerative disease research and treatment, the medical community must embrace AI with a balanced approach that maximizes its benefits while mitigating its risks. Let us move forward with cautious optimism, recognizing that AI, while not a panacea, holds the potential to significantly alter the landscape of neurodegenerative disease management for the better.

## Declaration of competing interest

The authors declare that they have no known competing financial interests or personal relationships that could have appeared to influence the work reported in this paper.

